# Protocol for mapping murine transcription factor interactomes and composite motifs combining affinity purification mass spectrometry and ChIP-seq

**DOI:** 10.1016/j.xpro.2025.104184

**Published:** 2025-11-04

**Authors:** Anna Gabele, Mert Cihan, Maximilian Sprang, Matthias Klein, Assel Nurbekova, Karolina Romaniuk, Niels Lemmermann, Stefan Tenzer, Miguel A. Andrade-Navarro, Tobias Bopp, Ute Distler

**Affiliations:** 1 Institute of Immunology, University Medical Center of the Johannes Gutenberg University Mainz, 55131 Mainz, Germany; 2 Research Center for Immunotherapy, University Medical Center of the Johannes Gutenberg University Mainz, 55131 Mainz, Germany; 3 Preventive Cardiology and Preventive Medicine, Center for Cardiology, University Medical Center of the Johannes Gutenberg University Mainz, 55131 Mainz, Germany; 4 Institute of Organismic and Molecular Evolution, Faculty of Biology, Johannes Gutenberg University Mainz, 55128 Mainz, Germany; 5 Institute for Virology, University Medical Center of the Johannes Gutenberg University Mainz, 55131 Mainz, Germany; 6 Institute of Virology, Medical Faculty, University Bonn, 53127 Bonn, Germany; 7 German Cancer Research Center (DKFZ), 69120 Heidelberg, Germany; 8 Immunoproteomics Unit, Helmholtz-Institute for Translational Oncology (HI-TRON) Mainz, 55131 Mainz, Germany

**Keywords:** bioinformatics, cell Biology, Genomics, ChIP-seq, Immunology, proteomics, mass spectrometry

## Abstract

Mass spectrometry (MS)-based approaches have significantly advanced our ability to study protein interaction networks in an unbiased manner. Here, we present a protocol that uses affinity purification (AP)-MS to identify interaction partners of a biotinylated transcription factor of interest, isolated from primary murine T cells. The resulting interactome data are integrated with motif analyses from chromatin immunoprecipitation sequencing (ChIP-seq) experiments. This combined approach facilitates the concurrent identification of protein interactors and composite DNA motifs, with each dataset corroborating the findings of the other.

For complete details on the use and execution of this protocol, please refer to Gabele et al*.*^1^

## Highlights

• A protocol to identify transcription factor complexes
• Steps for affinity purification mass spectrometry interactome analysis
• Integration of interactomics and ChIP-seq for the identification of composite motifs
• Guidance on combinatorial transcription factor motif analysis

## Abstract (editor highlights)

Publisher’s note: Undertaking any experimental protocol requires adherence to local institutional guidelines for laboratory safety and ethics.

## Graphical abstract

**Figure:**
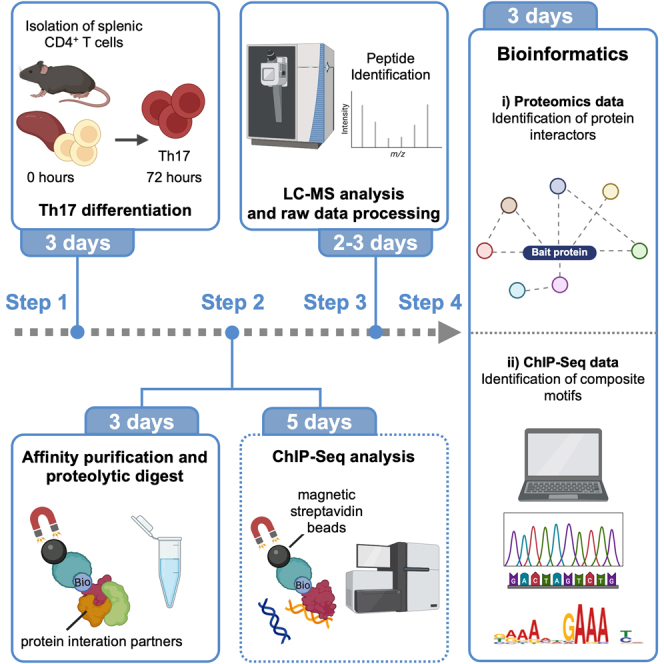

## Before you begin

### Background

Interactions among proteins and their crosstalk with other biomolecules are fundamental to virtually all cellular and physiological activities. Protein-protein interactions (PPIs) are essential for a wide range of biological processes, including signal transduction, gene regulation, cell differentiation, and proliferation. Aberrant PPIs are often associated with diseases, such as cancer or infectious and neurodegenerative diseases.^2^^,^^3^^,^^4^^,^^5^ Hence, the investigation of PPIs not only enhances our understanding of molecular disease mechanisms, but also supports the development of novel treatment strategies aimed at correcting or modulating dysfunctional PPIs.^2^

Over the past two decades, multiple mass spectrometry (MS)-based techniques, such as affinity purification (AP), proximity labeling, crosslinking, and co-fractionation MS, have evolved and greatly contributed to the depth and scale of protein interactome analysis. AP-MS is a robust and powerful approach to study PPIs with high specificity. In a typical AP-MS experiment, the protein of interest (bait) is enriched along with its interactors (prey) from a complex mixture using an antibody or another affinity matrix, that is directed either against the bait itself or against an affinity tag coupled to the target protein.^6^ The transgenic ROSA26^BirA^ mouse strain introduced by Driegen *et al.*^7^ ubiquitously expresses the bacterial BirA protein biotin ligase from the ROSA26 locus. BirA specifically biotinylates a short peptide sequence, also referred to as Avi-tag.^7^^,^^8^ Efficient *in vivo* biotinylation of a protein of interest can be achieved by crossbreeding ROSA26^BirA^ mice with any transgenic mouse strains expressing an Avi-tagged protein of interest. Using streptavidin-coupled affinity matrices, biotinylated proteins and their interactors can be isolated and subsequently analyzed by MS.^9^

In addition to the characterization of PPIs, this approach also enables the identification of target genes regulated by a transcription factor (TF) of interest applying streptavidin-mediated chromatin precipitation coupled with deep sequencing (Bio-ChIP-seq). ChIP-seq analysis combines the isolation of genomic regions, bound by a DNA-binding protein, with next-generation sequencing (NGS) technology to map specific genomic binding sites. Additionally, this method allows the identification of enriched binding motifs for transcriptional regulatory network analysis.^10^^,^^11^ Identifying co-binding motifs, particularly those in close proximity, can reveal potential synergistic or competitive interactions between TFs.^12^ Double-motif analysis has been used to distinguish between transcription factors that bind cooperatively and those that bind mutually exclusively, revealing functional binding dependencies that are not apparent from single motif analysis alone.^13^ It enables the detection of enriched motif pairs with defined spacing and orientation biases, which are indicative of direct physical interactions or coordinated DNA binding.^14^ Incorporating this approach into ChIP-seq workflows supports the systematic investigation of combinatorial transcriptional regulation and aids in the validation of predicted PPIs in a genomic context.

Our protocol provides detailed instructions for the isolation of a biotinylated TF of interest and its interactors as well as a guideline for combined motif analysis from (Bio-)ChIP-seq data to integrate and validate novel findings from PPI experiments. We describe the steps for a label-free AP-MS approach, including the enrichment of the target protein, MS sample preparation, and the detection of protein interaction partners by subsequent liquid chromatography (LC)-MS analysis. In addition, the protocol provides a step-by-step description on how to detect composite motifs in Bio-ChIP-seq data leveraging and integrating the information derived from both analytical techniques, i.e., LC-MS and ChIP-seq analysis. The study of Gabele *et al.*^1^ focuses on interacting proteins as well as DNA-binding regions of enzymatically, *in vivo* biotinylated Interferon Regulatory Factor 4 (IRF4) in primary T-cells. The described approach can be easily applied to any other protein of interest or cell type; however, it may require specific adjustments at particular steps in the protocol (which are highlighted in the following step-by-step description of the workflow).

### Innovation

The present protocol introduces an integrated workflow that combines label-free AP-MS with Bio-ChIP-seq motif analysis. Whereas PPIs or DNA-binding events are often studied in isolation, this approach enables the simultaneous identification of transcription factor interaction networks and composite DNA-binding motifs, thereby allowing cross-validation of findings from both analytical strategies.

### Institutional permissions

All animals were bred and housed at the Translational Animal Research Center (TARC) of the Johannes Gutenberg University of Mainz (Mainz, Germany) according to institutionally approved protocols. Animal experiments were performed under the supervision of authorized investigators in accordance with the European Union Normative for Care and Use of Experimental Animals with all relevant ethical regulations.

Prior to any research using transgenic mouse models, please refer to the local and country-specific guidelines and, if required, acquire permission from the relevant institutions.

### Sample preparation of biotinylated target protein for affinity purification

For the step-by-step protocol presented here, a biotinylated TF of interest is required as bait. Generally, any biotinylation approach that fits the experimental setup can be used.

In the study by Gabele *et al.*,^1^ an *in vivo* biotinylation approach using a transgenic mouse model was applied to selectively biotinylate the TF IRF4. To obtain endogenously biotinylated IRF4, we crossed the transgenic ROSA26^BirA^ mouse strain with transgenic mice expressing Avi-tagged IRF4 under the control of the endogenous *Irf4* promoter (IRF4^Avi-tag^ mice). ROSA26^BirA^ mice (strain *Gt(ROSA)26*^*Sortm1.1(birA)Mejr*^) can be purchased from Jackson Laboratory (https://www.jax.org/strain/010920). IRF4^Avi-tag^ mice (BAC transgenic mice; strain C57BL/6J-*Irf4*^*em1Bop*^) were obtained from Cyagen (https://www.cyagen.com), which provides transgenic mouse services with custom-tailored genetic modifications.

**Figure 1:**
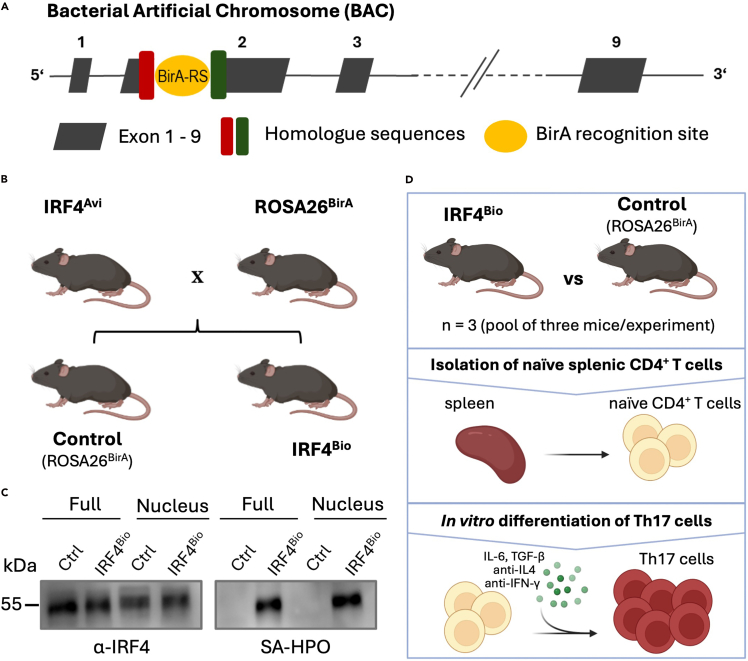
Generation of the IRF4^Bio^ mouse strain and *in vitro* differentiation of primary T cells into Th17 cells (A) Schematic overview of the bacterial artificial chromosome (BAC) transgene production for the generation of the IRF4^Bio^ mouse strain. (B) Cross-breeding of animals: IRF4^Avi^ mice, containing the BAC transgene, are crossed with ROSA26^BirA^ mice, ubiquitously expressing BirA ligase under the ROSA26 promoter, resulting in offsprings that express *in vivo* biotinylated IRF4 (IRF4^Bio^). (C) Western blot analyses of full cellular lysates and nuclear extracts demonstrate successful *in vivo* biotinylation of IRF4 (left, anti-IRF4 antibody; right, horseradish peroxidase-conjugated streptavidin [SA-HPO]). (D) Workflow for *in vitro* differentiation of primary Th17 cells.

For the generation of a primary T cell subset, naïve CD4^+^ T cells from IRF4^Bio^ and control mice (ROSA26^BirA^) were isolated from the spleen of the animals and differentiated *in vitro* for three days under Th17-skewing conditions using a cytokine cocktail containing TGF-ß, IL-6, anti-IFN-γ and anti-IL4 (Figure 1). All details on the establishment of the mouse model and the *in vitro* differentiation of primary T cells can be found in the associated research paper.^1^

### Chromatin immunoprecipitation coupled with sequencing

Prior to the (combined) motif analysis presented in this protocol, a successful ChIP-seq analysis is required, for which several protocols exist and are described in detail elsewhere.^15^^,^^16^^,^^17^^,^^18^

A Bio-ChIP-seq approach was used for the analysis presented in this protocol. In brief, fully *in vitro* differentiated Th17 cells with an endogenously biotinylated TF (IRF4) were generated, fixed and the cellular lysate subjected to ChIP-seq analysis. IRF4 and bound DNA were isolated using streptavidin-coated magnetic beads. After several extensive washing steps, proteins and RNA were enzymatically eliminated. The remaining chromatin fragments were cleaned and concentrated for sequencing. The software EaSeq^19^ was used for analysis of ChIP-seq data and the respective called genomic peak regions were exported as Excel file and subjected to bioinformatics motif analysis.^1^

## Key resources table

**Table:** 

REAGENT or RESOURCE	SOURCE	IDENTIFIER
**Chemicals, peptides, and recombinant proteins**		

Acetic acid, ROTIPURAN 100%, p.a.	Carl Roth	Cat#: 3738.2
Dithiobis[succinimidyl propionate] (DSP)	Thermo Fisher Scientific	Cat#: 22585
cOmplete protease inhibitor cocktail (PIC)	Roche	Cat#: 11697498001
Dimethyl sulfoxide (DMSO) ROTISOLV ≥99,99%, headspace grade	Carl Roth	Cat#: HN47.1
Magnesium chloride (MgCl_2_)	Carl Roth	Cat#: KK36.1
Sodium chloride (NaCl) ≥99,5%, p.a., ACS, ISO	Carl Roth	Cat#: 3957.2
Igepal CA-630	Sigma-Aldrich	Cat#: I8896-100ML
Sodium deoxycholate	Merck	Cat#: 30970-25G
Sodium dodecyl sulfate (SDS)	Carl Roth	Cat#: 8029.4
Sodium dihydrogen phosphate dihydrate ≥99%, p.a	Carl Roth	Cat#: T879.3
Sodium chloride ≥99% (NaCl, used for PBS)	Carl Roth	Cat#: 0601.2
Sodium hydroxide ≥98%, Ph. Eur., USP, BP, in pellets	Carl Roth	Cat#: P031.3
Tris PUFFERAN ≥99.9%, p.a.	Carl Roth	Cat#: 4855.2
TRIS hydrochloride PUFFERAN ≥99.9%, p.a. (Tris-HCl)	Carl Roth	Cat#: 9090.3
Biotin	Sigma-Aldrich	Cat#: B4501-1G
Iodoacetamide (IAA) BioUltra	Sigma-Aldrich	Cat#: I1149-5G
1,4-dithiothreitol (DTT) ≥99%, p.a.	Carl Roth	Cat#: 6908.1
Ammonium hydrogen carbonate (AMBIC) ≥99%, p.a.	Carl Roth	Cat#: T871.1
Roti-PreMix PBS	Carl Roth	Cat#: 0890.1
Trypsin Gold, mass spectrometry grade	Promega	Cat#: V5280
Nuclease-free water	QIAGEN	Cat#: 0890.1
ROTISOLV acetonitrile ≥99,98%, Ultra LC-MS	Carl Roth	Cat#: HN40.1
ROTISOLV water, Ultra LC-MS (LC-MS grade water)	Carl Roth	Cat#: HN43.1
Ethanol (pure)	PanReac AppliChem	Cat#: 141086.1211
Formic acid ROTIPURAN ≥99%, LC-MS grade	Carl Roth	Cat#: 1EHK.1

**Deposited data**		

Mouse reference genome (mm10)	UCSC Genome Browser	http://hgdownload.soe.ucsc.edu/goldenPath/mm10/
IRF4 ChIP-seq data	NCBI GEO	GSE240979
Mass spectrometry proteomics data	ProteomeXChange/jPOST	PXD044298/ JPST002260 (murine data)
TRANSFAC	TRANSFAC Database^20^	https://genexplain.com/transfac/
R scripts used for statistical analysis	GitHub/Zenodo	https://github.com/Muedi/IRF4-in-Treg-and-Th17; https://doi.org/10.5281/zenodo.14643315

**Software and algorithms**		

R (≥4.1.0)	R Project	https://cran.r-project.org
readxl (≥1.4.3)	Wickham et al.^21^	https://readxl.tidyverse.org
doParallel (≥1.0.17)	Folashade et al.^22^	https://cran.r-project.org/package=doParallel
foreach (≥1.5.2)	Folashade et al.^23^	https://cran.r-project.org/package=foreach
bedtools (≥2.30.0)	Quinlan and Hall^24^	https://bedtools.readthedocs.io
samtools (≥1.13)	Danecek et al.^25^	http://www.htslib.org/
TRANSFAC Match tool	geneXplain^20^	https://genexplain.com/transfac/
dplyr (≥1.1.4)	Wickham et al.^26^	https://dplyr.tidyverse.org
stringr (>1.5)	Wickham et al.^26^	https://stringr.tidyverse.org/
arrow (>20)	Richardson et al.^27^	https://arrow.apache.org/docs/r/
limma (>3.64)	Ritchie et al.^28^	https://bioinf.wehi.edu.au/limma/
impute (>1.8)	Hastie et al.^29^	https://www.bioconductor.org/packages/release/bioc/html/impute.html

**Other**		

Dynabeads M-280	Thermo Fisher Scientific	Cat#: 11205D
Sera-Mag carboxylate-modified magnetic beads & SpeedBeads	Cytiva	Cat#: 65152105050250 Cat#: 45152105050250
Magnetic rack	Cell Signal	Cat#: 7071S
Bioruptor Plus sonication device	HOLOGIC Diagenode	Cat#: B01020014
Thermo Scientific Exploris 480 mass spectrometer or any other state-of-the art mass spectrometer	Thermo Fisher Scientific	N/A
Ultimate 3000 RSLCnano LC system	Thermo Fisher Scientific	N/A
PEPMAP Neo C18 5 μm, 300 μm × 5 mm trap column	Thermo Fisher Scientific	Cat#: 174500
HSS-T3 C18 1.8 μm, 75 μm × 250 mm analytical LC column	Waters	Cat#: 186008818

## Materials and equipment

### Solutions to be prepared in advance

• 0.1% (v/v) Formic acid: Mix 1 μL formic acid with 999 μL LC-MS grade water.

**CRITICAL:** Formic acid is flammable and volatile. It causes severe burns. Handle it in a fume hood and wear appropriate gloves and safety glasses.

• 70% (v/v) Ethanol: Mix 70 μL ethanol with 30 μL LC-MS grade water.

**CRITICAL:** Ethanol is highly flammable.

• 10% (v/v) Igepal: Add 1 mL Igepal to 9 mL of nuclease-free water. Store at 4°C.

**CRITICAL:** Igepal causes skin irritation and severe eye damage. Wear gloves and safety glasses.

• 50× Protease Inhibitor Cocktail (PIC): Dissolve 1 tablet PIC in 1 mL LC-MS grade water and aliquot. Store at −20°C.
• Crosslinker stopping solution (1 M Tris, pH 7.5): Dissolve 1.58 g Tris in 10 mL sterile 1× PBS. Adjust the pH to 7.5 and store at 4°C.

**Table:** Hypotonic buffer

Reagent	Final concentration	Amount
Tris-HCl	20 mM	157.6 mg
NaCl	10 mM	29.2 mg
MgCl_2_	3 mM	14.3 mg
ddH_2_O (dd, double-distilled)	N/A	Add to 50 mL

***Note:*** Adjust pH to 7.5 using NaOH before adding ddH_2_O to a total of 50 mL. Store at 4°C up to one month.

**Table:** RIPA buffer

Reagent	Final concentration	Amount
Tris	25 mM	151 mg
NaCl	150 mM	438 mg
Igepal	1% (v/v)	500 μL
Sodium deoxycholate	1% (w/v)	500 mg
SDS	0.1% (w/v)	5 mg
LC-MS grade water	N/A	Add to 50 mL

***Note:*** Dissolve Tris and NaCl in 40 mL of LC-MS grade water and adjust pH to 7.5 before adding detergents using HCl. In a final step, fill up with LC-MS grade water to a total of 50 mL. Store at 4°C for 1–2 weeks. For long-term storage, freeze aliquots (e.g., 1 mL each) at −20°C.

**CRITICAL:** SDS is corrosive and flammable, use dust mask type N95 (US), wear appropriate gloves and safety glasses. Igepal causes skin irritation and severe eye damage. Wear gloves and safety glasses.

Phosphate-Buffered Saline (PBS) 10×, 5 liters.

**Table:** PBS buffer (pH 8.3)

Reagent	Final concentration	Amount
NaH_2_PO_4_	0.1 M	78 g
NaCl	1.3 M	402 g
Ultrapure water	N/A	Add to 5L

***Note:*** Dissolve the salts in 4.5L of water. Adjust pH of the 10× PBS to 6.6 using a 10 M NaOH solution before adding water to a final volume of 5L. 1× PBS is prepared by diluting 10× PBS with ultrapure water at a ratio of 1:10. Adjust to pH 8.3 using a 10 M NaOH solution. Autoclave 1× PBS. Store at 25°C for up to six months. Alternatively, premixed PBS can be used to generate a 1× PBS solution ready-to-use (e.g., ROTIPreMix PBS, see also key resources table).

**Table:** Protein elution buffer (SDS-biotin buffer)

Reagent	Final concentration	Amount
Tris	10 mM	6.1 mg
Biotin	10 mM	12.2 mg
SDS	1% (w/v)	50 mg
LC-MS grade water	N/A	Add to 5 mL

***Note:*** Adjust pH to 7.5 using HCl before adding LC-MS grade water to a total of 5 mL. Store at 4°C for up to one month.

**CRITICAL:** SDS is corrosive and flammable, use dust mask type N95 (US), wear appropriate gloves and safety glasses.

• SP3 bead stock solution (20 μg solids/μL): Combine 20 μL of Sera-Mag carboxylate-modified magnetic particles (hydrophobic, provided at a concentration of 50 μg/μL by the vendor) and 20 μL of Sera-Mag Speed-Bead carboxylate-modified magnetic particles (hydrophilic, 50 μg/μL) in a 1.5 mL-tube. Add 160 μL of LC-MS grade water and mix. Place the tube with beads on a magnetic rack and remove supernatant after beads have settled. Off the rack, add 200 μL of LC-MS grade water and mix. Repeat wash steps two further times. Recover beads in 100 μL of LC-MS grade water.

***Note:*** SP3 bead stock can be stored at 4°C for up to 1 month.

• Trypsin stock solution: Dissolve lyophilized trypsin in 50 mM acetic acid prepared in LC-MS grade water to a final concentration of 1 μg/μL. If not directly used for proteolytic digestion, aliquot and store at −80°C.

**CRITICAL:** Acetic acid is flammable. It causes severe burns. Wear protective gloves and safety glasses.

**Table:** Wash buffer I (RIPA buffer containing 0.35% (w/v) SDS)

Reagent	Final concentration	Amount
Tris	25 mM	151 mg
NaCl	150 mM	438 mg
Igepal	1% (v/v)	500 μL
Sodium deoxycholate	1% (w/v)	500 mg
SDS	0.35% (w/v)	175 mg
LC-MS grade water	N/A	Add to 50 mL

***Note:*** Dissolve Tris and NaCl in 40 mL of LC-MS grade water and adjust pH to 7.5 using HCl before adding detergents. In a final step, fill up with LC-MS grade water to a total of 50 mL. Store at 4°C for 1–2 weeks. For long-term storage, freeze aliquots (e.g., 1 mL each) at −20°C.

**CRITICAL:** SDS is corrosive and flammable, use dust mask type N95 (US), wear appropriate gloves and safety glasses. Igepal causes skin irritation and severe eye damage. Wear gloves and safety glasses.

• Wash buffer II (25 mM Tris): Dissolve 151 mg Tris in 50 mL LC-MS grade water. Adjust pH to 7.5 using HCl and store at 4°C.

### Solutions to be prepared on the day of the experiment

• 20 mM Dithiobis[succinimidylpropionate] (DSP, crosslinker stock solution): Dissolve 4.044 mg DSP just before use in 500 μL DMSO.

***Note:*** DSP is recommended to be stored at 4°C–8°C. As DSP is moisture-sensitive, equilibrate vial for 30 min at room temperature before opening. Prepare crosslinker stock solution just before usage and keep at room temperature.

**CRITICAL:** DSP causes respiratory and skin irritation as well as serious eye damage. Wear gloves and safety glasses. Avoid breathing dust. DMSO enhances dermal absorption. Wear appropriate gloves.

• 0.75 mM DSP (crosslinker working solution): Dilute 20 mM DSP stock solution in PBS, pH = 8.3 to a final concentration of 0.75 mM DSP.

***Note:*** Prepare a master mix for all samples of one experiment to make handling easier and faster.

**CRITICAL:** DSP causes respiratory and skin irritation as well as serious eye damage. Wear gloves and safety glasses. Avoid breathing dust.

• 50 mM Ammonium bicarbonate (NH_4_HCO_3_): Dissolve 39.53 mg NH_4_HCO_3_ in 10 mL LC-MS grade water.
• 450 mM 1,4-Dithiothreitol (DTT): Dissolve 693.9 mg DTT in 10 mL LC-MS grade water.

**CRITICAL:** DTT is harmful if swallowed, and it is an eye and skin irritant. Wear gloves and safety glasses.

• 2% (v/v) Dimethyl sulfoxide (DMSO): Mix 2 μL DMSO with 98 μL LC-MS grade water.
• 800 mM Iodoacetamide (IAA): Dissolve 1.48 g IAA in 10 mL LC-MS grade water.

**CRITICAL:** IAA is toxic if swallowed, and should be handled in a fume hood. Wear appropriate gloves and safety glasses.

• Trypsin working solution: Fill up 5 μL of trypsin stock solution (1 μg/μL) to a final volume of 62.5 μL using 50 mM NH_4_HCO_3_ resulting in a final concentration of 0.08 μg trypsin/μL.

## Step-by-step method details

### Chemical crosslinking and isolation of cell nuclei

**Timing: 2–3 h depending on the number of samples**

The steps below describe how to crosslink protein-protein complexes in cells and how to isolate cell nuclei. Using a crosslinker, PPIs can be preserved *in situ* prior to cell lysis and nuclear extraction while cells are still intact. Moreover, more stringent wash conditions can be applied during AP reducing unspecific background, while concomitantly preserving weak interactions.^1^^,^^30^

***Note:*** The details below describe the crosslinking of a nuclear TF in primary suspension cells using the reversible, membrane-permeable crosslinker DSP. If another crosslinker is used, please read the product information and follow the manufacturer’s instructions on the use of the respective crosslinker.

**CRITICAL:** Each pulldown experiment requires an *in vivo* biotinylated sample and a control sample. We recommend to use samples isolated by the same protocol from the parental transgenic ROSA26^BirA^ mouse strain as control samples. It is recommended to perform at least three biological replicates.

1. Harvest primary, *in vitro* generated T cells (suspension cells).
  ***Note:*** This step must be adjusted if working with adherent cells. Please proceed with your standard protocol for harvesting adherent cells.
  a. Re-suspend and transfer cells into a 50 mL tube.
  b. Wash cell culture plate with pre-warmed PBS to collect remaining cells. Combine with cells harvested in a.
  c. Wash collected cells twice (10 min at 630 × g at 4°C) with cold PBS to remove any remaining medium.
  d. Count cells and adjust cell numbers between biotinylated samples and controls.
    ***Note:*** The optimal cell number per pulldown experiment is dependent on the expression level of your protein of interest and cell size. We used around 3 × 10^7^ T cells. Here, we briefly summarize buffer and bead volumes used in the next steps for cell lysis and AP, which have to be adjusted depending on the cell number used:

**Table:** 

Cell No.	Crosslinking		Isolation of nuclei		Affinity purification		
	DSP working solution	Crosslinker stopping solution	Hypotonic buffer	10% (v/v) Igepal	RIPA buffer (nuclei lysis)	Strept-avidin beads	Re-suspension of beads after RIPA wash
1 × 10^7^	500 μL	10 μL	1 mL	50 μL	30 μL	15 μL	60 μL
    Increase volumes for higher cell numbers, e.g., for 2 × 10^7^ use 1 mL of DSP working solution, 20 μL of stopping solution etc.
2. Crosslink interacting proteins/protein complexes.
  **CRITICAL:** The optimal concentration of crosslinker must be adjusted correspondingly for each cell type and crosslinker type in advance.^30^ Always refer to manufacturer’s instructions for the use of the crosslinker.
  a. Discard PBS after last washing step.
  b. Re-suspend cells in 0.75 mM DSP working solution.
    ***Note:*** Use 500 μL DSP working solution per 1 × 10^7^ cells. Adjust volume for the respective cell count and prepare a master mix for all samples of one experiment. Discard any unused crosslinker.
  c. Incubate cells at room temperature for 30 min. Invert tube at regular intervals.
  d. Add crosslinker stopping solution to a final concentration of 20 mM Tris to stop crosslinking reaction (e.g., 10 μL per 500 μL DSP working solution).
  e. Incubate cells at room temperature for 15 min. Invert tube at regular intervals.
  f. Wash cells twice (10 min at 630 × g at 4°C) with cold PBS.
3. Isolate cell nuclei.
  a. Immediately before use, add 50× PIC stock solution to the hypotonic buffer at a 1:50 dilution to prepare 1× PIC-supplemented hypotonic buffer.
    ***Note:*** Use 1 mL hypotonic buffer per 1 × 10^7^ cells. Adjust volume for the respective cell count and prepare a master mix for all samples of one experiment.
  b. Discard PBS after last washing step.
  c. Re-suspend cells in hypotonic buffer supplemented with 1× PIC.
  d. Incubate cells on ice for 12 min.
    **CRITICAL:** The incubation time has to be adjusted for each cell type in advance. A too short incubation time does not remove contaminating cytoplasmic proteins while a too long incubation time results also in the disruption of the nuclear membrane and, therefore, in the loss of nuclear proteins.
  e. Add 10% (v/v) Igepal to a final concentration of 0.5% (v/v) and vortex for 10 s to disrupt cell membranes.
  f. Pellet nuclei (10 min at 850 × g at 4°C).
  g. Wash cell nuclei twice (10 min at 850 × g at 4°C) with 10 mL cold PBS.
  h. For the final wash step, transfer cell nuclei into fresh 1.5 mL LoBind protein Eppendorf tubes.
  i. Carefully remove and discard the supernatant after the last wash and continue next steps with the dry cell nuclei pellet.
    **Pause point:** Samples can either be stored at −80°C until further processing or directly subjected to immunoprecipitation.
    ***Optional:*** Save supernatant after cell lysis (step 3f), which contains cytoplasmic proteins, to assess purity of cytoplasmic fraction and potential nuclear contamination by western blotting.

### Affinity purification of protein complexes

**Timing: 2 days**

The steps below describe the isolation of the endogenous biotinylated target protein with its interacting proteins for mass spectrometric analysis using magnetic streptavidin-coated beads.

4. Pre-cool Bioruptor Plus at 4°C.
5. Lyse cell nuclei.
  **CRITICAL:** As the optimal lysis and binding conditions may depend on your target protein, it is recommended to compare different lysis and binding buffers in advance. For example, in the case of the TF IRF4, good results were achieved using the μMACS FactorFinder Kit by Miltenyi, which has been discontinued. RIPA buffer represents a good non-commercial alternative for nuclear lysis and protein binding.
  Supplement RIPA lysis buffer to a final amount of 1× PIC directly before use.
  The volumes below correspond to 1 × 10^7^ cell nuclei and have to be adjusted if cell numbers differ.
  a. If cell nuclei were stored at −80°C, gently thaw samples on ice.
  b. Add 30 μL RIPA buffer to cell nuclei and mix gently.
  c. Sonicate cell nuclei twice for 5 min each at 4°C (low, 30 s ON/ 30 s OFF) in the Bioruptor Plus to disrupt nuclear membrane and DNA. Briefly invert and spin down the lysate between the sonication cycles.
  d. Centrifugate samples for 6 min at 14,850 × *g* at 4°C.
  e. Transfer the supernatant, containing nuclear proteins, to a new 1.5 mL protein LoBind Eppendorf tube. Store lysate on ice until the next steps are completed.
    ***Optional:*** Take a small amount of the lysate as an input control for western blot analysis.
6. Prepare magnetic streptavidin-coated beads.
  a. Resuspend magnetic streptavidin-coated beads before use.
  b. Add 15 μL beads per sample into a new 1.5 mL protein LoBind Eppendorf tube.
    ***Note:*** The beads for all samples of an experiment can be prepared and washed in one mix.
  c. Place tube on magnet and discard storage buffer once beads have settled.
  d. Wash beads with RIPA buffer on a rotating shaker for 10 min at 21°C–24°C.
  e. Spin tubes briefly down and place them on a magnetic rack. Once the beads have settled, discard supernatant.
  f. Re-suspend beads in 60 μL RIPA buffer supplemented with 1× PIC per sample.
7. Add resuspended beads to the nuclear lysates.
8. Incubate samples rotating over night at 4°C.
9. Affinity purification of biotinylated protein of interest.
  a. Spin tubes briefly down and place them on a magnetic rack for ∼2 min.
  b. Discard supernatant once beads have settled and supernatant becomes clear.
  c. Re-suspend proteins in 1 mL wash buffer I and wash sample rotating for 10 min at 21°C–24°C.
  d. Repeat the washing step c two more times. Discard the supernatant after each washing step.
  e. Re-suspend proteins in 1 mL wash buffer II after the third washing step.
  f. Wash samples three times with wash buffer II as already described for wash buffer I (rotating for 10 min at 21°C–24°C).
  g. Discard the supernatant after a total of six washing steps.
  h. Resuspend the magnetic beads in 50 μL SDS-biotin buffer.
  i. Incubate the samples for 5 min at 95°C, shaking at 350 U/min, to elute the proteins from the beads.
  j. Place the samples on a magnetic rack for 2 min and transfer the supernatant into a new 1.5 mL protein LoBind Eppendorf tube.

***Optional:*** Take a small amount of the eluate as a control for western blot analysis.

**Pause point:** Samples can either be stored at −80°C until further processing or directly subjected to proteolytic digestion.

### Proteolytic digestion

**Timing: 1.5 days**

The steps below describe the proteolytic digestion of proteins using single-pot solid-phase-enhanced sample preparation (SP3).^31^^,^^32^^,^^33^ SP3 was introduced in 2014 by Hughes *et al.*^31^ as a streamlined single-tube approach that integrates protein and peptide clean-up with tryptic digestion, thereby reducing sample handling and minimizing sample loss. SP3 utilizes carboxylate functionalized paramagnetic beads to capture proteins by a hydrophilic interaction mechanism enabling the efficient removal of different agents such as chaotropes and detergents, which impair downstream MS-analysis. This is particularly relevant for the processing of the affinity-purified samples in the present protocol, as wash and elution buffers contain detergents.

***Note:*** The volumes below correspond to 50 μL input volume. Adjust accordingly if the input volume varies or different stock concentrations of DTT and IAA are used.

10. Cleave DSP crosslinker and reduce disulfide bonds.
  a. If samples were stored at −80°C, gently thaw them.
  b. Add 5.5 μL DTT (450 mM) to the eluted proteins to cleave DSP crosslinker and reduce disulfide bonds in samples with a final concentration of 50 mM DTT.
  c. Incubate samples for 30 min at 37°C.
    ***Note:*** For alternative, reversible crosslinkers, check manufacturer’s instructions for recommended crosslinker cleavage.
  d. Increase the temperature to 45°C and incubate for 10 min.
11. Alkylate free cysteine residues.
  a. Add 3.5 μL IAA (800 mM) to alkylate samples at a final concentration of 50 mM IAA.
  b. Incubate for 30 min at 21°C–24°C in the dark.
12. Quench alkylation reaction by adding 2.6 μL DTT (450 mM) for a final concentration of 20 mM DTT.
13. Add 2 μL of the pre-washed SP3 beads to each sample (see also paragraph “solutions to be prepared in advance”).
14. Add 148.4 μL of acetonitrile (ACN) to the sample, resulting in a final concentration of 70% (v/v) ACN.
15. Incubate the samples for 20 min at 21°C–24°C. Mix samples after 10 min as soon as sedimentation of the protein-bead aggregates is observed.
16. Spin tubes briefly down and place samples on a magnetic rack for at least 2 min until all beads have settled.
17. Once the beads have fully settled and supernatant becomes clear, carefully remove and discard supernatant.
18. Add 200 μL 70% (v/v) ethanol to each sample.
19. Incubate each sample for 30 s on the magnetic rack and discard the supernatant afterward.
20. Repeat steps 18 and 19 one more time.
21. Discard the supernatant.
22. Add 180 μL ACN to each sample.
23. Incubate each sample for 30 s on the magnetic rack and discard the supernatant afterward. During this final ACN rinse, remove as much of the residual liquid as possible from the tube (<5 μL is optimal) to avoid carryover to the enzyme digestion steps.
24. Remove the tubes with opened lids from the magnetic rack and briefly air-dry for around 5 min to further minimize residual amounts of ACN. Complete evaporation is not necessary.
25. Re-suspend each sample in 5 μL trypsin working solution and incubate for 12–16 h at 37°C.

***Note:*** A trypsin-to-protein ratio of 1:25 (w/w) is recommended. The total protein amount in the affinity-purified samples depends on various factors, such as the expression level of the biotinylated bait, the cell type and number used for the AP-MS experiments, etc. In case of IRF4 isolated from around 3–4 × 10^7^ CD4^+^ T cells, we add a total amount of 0.4 μg of trypsin.

26. On the next day, resuspend samples and add 95 μL ACN to each sample for a final concentration of 95% (v/v) ACN.
27. Vortex samples immediately.
28. Incubate the samples for 20 min at 21°C–24°C to aggregate peptides and beads. Mix samples after 10 min as soon as sedimentation of the protein-bead aggregates is observed.
29. Spin tubes briefly down and place samples on a magnetic rack for at least 2 min until all beads have settled.
30. Once supernatant becomes clear, discard supernatant.

***Optional:*** Transfer the supernatant into a new LoBind 1.5 mL Eppendorf tube for an additional peptide-capture and clean-up step to increase peptide yield. Add 2 μL of SP3 beads to the collected supernatants. Incubate the samples for 20 min at 21°C–24°C. Mix samples after 10 min. Spin tubes briefly down and place samples on a magnetic rack for at least 2 min until all beads have settled. Discard supernatant. Then follow steps 32 and 33. Combine resuspended magnetic beads with the original sample after step 32 (prior to sonication, step 33).

31. Add 180 μL ACN to the beads, place the samples on a magnetic rack, and discard the supernatant.
32. Let residual ACN evaporate for about 5 min. Resuspend beads in 10 μL freshly prepared 2% (v/v) DMSO to elute peptides from beads.
33. Sonicate samples for 1 min to improve peptide recovery.
34. Centrifuge the samples for 2 min at 16,200 × *g* and 4°C.
35. Place samples on a magnet rack and transfer the supernatant into a glass vial for MS analysis. Acidify the samples by adding 10 μL of 0.1% (v/v) formic acid.

**CRITICAL:** Ensure that no beads are transferred with the supernatant.

**Pause point:** Samples can either be stored at −80°C until further processing or directly analyzed by LC-MS.

### Liquid chromatography-mass spectrometry analysis

**Timing: ∼1 h/sample**

The following section briefly outlines how analyze the samples by LC-MS analysis. In general, any high-resolution, state-of-the-art LC-MS instrument platform can be used for LC-MS analysis. In the study by Gabele *et al.*,^1^ samples were analyzed on an Ultimate 3000 RSLCnano LC system (Thermo Fisher Scientific) coupled to an Orbitrap Exploris 480 instrument platform (Thermo Fisher Scientific).

36. Perform LC-MS analysis of the tryptic digests.
  a. Separate tryptic peptides by reverse-phase LC.
    ***Note:*** For peptide separation, we favor the use of commercial reverse-phase C18 HPLC columns with the following dimensions: 75 μm inner diameter (ID) x 25 cm length, particle size ≤ 1.8 μm (see key resources table). However, shorter columns and/or inner diameters of 150 μm can be used as well. Similarly, we favor a trap setup, where peptides are first loaded onto a PEPMAP100 C18 5 μm 0.3 × 5 mm trap column (Thermo Fisher Scientific) to extend the lifetime of the analytical column. Typically, we run 30–40 min gradients for the chromatographic separation of peptides, but shorter gradients should suffice as well.
  b. Analyze eluting peptides by MS.
    ***Note:*** For mass spectrometric analysis of eluting peptides, we favor data-independent acquisition (DIA) methods. We recommend the following settings on the Orbitrap Exploris 480 instrument platform: Spray voltage of 1.8 kV (may need to be adjusted depending on the emitter setup used), funnel RF level at 40, and heated capillary temperature at 275°C, full MS resolution set to 120,000 at *m/z* 200 and full MS automated gain control (AGC) target to 300% with a maximum injection time (IT) of 20 ms. Set mass range to *m/z* 345 – 1,250. Acquire fragment ion spectra with an AGC target value of 1000%. We recommend to program around 20 windows with varying sizes (adjusted to precursor density) and an overlap of 0.5 Th at a resolution of 30,000. IT can be determined automatically (“auto mode”). Fix HCD collision energy was at 27%. Acquire all data in profile mode using positive polarity.
    ***Note:*** It is beyond the scope of this protocol to exhaustively review all options for the LC-MS/MS analyses as these depend on the instrument platforms available. Here, we describe our preferences and provide some comments on the settings.

### LC-MS raw data processing and label-free quantification using DIA-NN

***Note:*** The steps below provide a brief description of raw data processing, identification and label-free quantification (LFQ) of peptides and proteins in DIA-NN^34^ for the affinity-purified samples. Other software tools such as Spectronaut,^35^ FragPipe^36^ and PECAN^37^ may be used for library-free DIA data processing. New versions of DIA-NN are released on a regular basis and can be freely downloaded from https://github.com/vdemichev/DiaNN. Detailed installation instructions as well as descriptions of individual search settings and parameters are provided on the GitHub repository from DIA-NN (readme).

37. Launch the DIA-NN software.
38. Select and load your raw data files into the software tool.
39. Select the FASTA file for the library-free DIA analysis containing the reference proteome of the species from which your samples originate. In case of murine samples, search the LC-MS dataset against a database comprising the reviewed entries of the *Mus musculus* reference proteome that can be downloaded from UniProt: https://www.uniprot.org/proteomes/UP000000589.
40. We recommend to use the “default settings” as preset in the DIA-NN GUI with the following adaptations for AP-MS experiments: i) match-between-runs (MBR) enabled, ii) cross-run normalization switched “off”, and iii) heuristic protein inference enabled.
41. Specify the path for your search outputs.
42. Run DIA-NN.
43. The resulting report file (.tsv format for DIA-NN versions <2.0, .parquet for DIA-NN versions 2.0 and higher) contains quantitative information about peptides and proteins.

### Installation of tools and packages for the bioinformatics analyses of interactome data and DNA-binding motifs

**Timing: 0.5 h**

The following sections describe how to identify statistically significant protein interactors of IRF4 from AP-MS experiments between two conditions. In addition, they detail how to integrate Bio-ChIP-seq data with motif annotation using immune-specific TRANSFAC profiles to uncover potential combinatorial transcriptional regulation in IRF4-bound regions. Here, IRF4 binding sites are cross-referenced with nearby TF motifs relevant to T cell identity. This allows to identify significant interactors that co-occur within double-motif contexts revealing potential cooperative binding.

Before running this analysis, install R (https://www.r-project.org/) and ensure all required R packages and tools are available. The following setup assumes R version ≥4.1.0 on a Unix-based system.

44. Install R packages.

install.packages(c("dplyr", "readxl", "doParallel", "foreach", "plyr","stringr","arrow"))

if (!require("BiocManager", quietly = TRUE)) install.packages("BiocManager")

BiocManager::install(c("limma", "impute"))

### Identification of statistically significant IRF4 interactors

**Timing: 1 day**

The steps below describe how to identify significant IRF4 interactors by comparing AP-MS data between conditions, e.g., samples containing the biotinylated protein of interest versus control samples, using limma.^28^ The input requires a .csv table with protein names in the first column, the sample intensities in the remaining columns. Include clear condition labels in the column names (e.g., “Bio” vs. “Ctrl”).

45. Filter and transform the DIA-NN output.

***Note:*** As indicated above DIA-NN generates a main output report (.tsv format for DIA-NN versions <2.0, .parquet for DIA-NN versions 2.0 and higher) containing precursor and protein identifications, quantitative values, as well as plenty of additional associated data. Exemplary code below extracts protein names and label-free quantification (LFQ) values from the DIA-NN report (.parquet file), listing only proteins identified by at least two peptides. Inspect the data and remove contaminants and reverse hits from the list.

library(arrow)

library(dplyr)

library(tidyr)

#Load DIA-NN output

df <- read_parquet("dia-nn_output.parquet")

#Count unique stripped sequences per protein

peptide_counts <- df %>% filter(!is.na(PG.MaxLFQ)) %>%

 mutate(Peptide = paste(Modified.Sequence, Precursor.Charge, sep = "_")) %>%

 distinct(Protein.Names, Stripped.Sequence) %>% count(Protein.Names, name = "NumPeptides")

#Filter for proteins with ≥2 unique stripped peptides

proteins_with_2plus <- peptide_counts %>% filter(NumPeptides >= 2)

#Summarize and reshape PG.MaxLFQ values

protein_matrix <- df %>% filter(Protein.Names %in% proteins_with_2plus$Protein.Names, !is.na(PG.MaxLFQ)) %>% group_by(Protein.Names, Run) %>% summarise(PG.MaxLFQ = mean(PG.MaxLFQ, na.rm = TRUE), .groups = "drop") %>% pivot_wider(names_from = Run, values_from = PG.MaxLFQ)

write.csv(protein_matrix,"input_APMS.csv",row.names = F)

46. Load filtered DIA-NN output and pre-process AP-MS data.

library(readr)

library(stringr)

library(impute)

library(limma)

# Maximum NAs allowed per group (e.g., Ctrl or Bio)

max_missing_values_per_condition <- 8

# Load data

df <- as.data.frame(read_csv("input_APMS.csv"))

rownames(df) <- df$Protein.Names

df$Protein.Names <- NULL # Remove identifier column

# Define conditions

conditions <- ifelse(str_detect(colnames(df), "Bio"), "Bio", "Ctrl")

# Filter proteins with too many missing values in any group

filter_missing <- function(data, conds, max_na) {

 keep <- apply(data, 1, function(row) {

 all(sapply(unique(conds), function(g) sum(is.na(row[conds == g])) <= max_na))

})

 data[keep, ]

}

df <- filter_missing(df, conditions, max_missing_values_per_condition)

# Log2 transformation

df <- log2(df + 1)

# Impute missing values (KNN)

df_imp <- impute.knn(as.matrix(df))$data

47. Perform limma analysis and save results.

# Design matrix and contrast

design <- model.matrix(∼0 + factor(conditions))

colnames(design) <- levels(factor(conditions))

contrast <- makeContrasts(Bio_vs_Ctrl = Bio - Ctrl, levels = design)

# Differential analysis with limma

fit <- lmFit(df_imp, design)

fit <- contrasts.fit(fit, contrast)

fit <- eBayes(fit)

# Extract results

results <- topTable(fit, number = Inf)

results$Protein.Names <- rownames(results)

write_csv(results, "results_limma.csv")

***Note:*** Quality control steps and imputation strategies depend on the experimental design and data quality, and may vary between datasets. Adjust filtering thresholds, transformation methods, or imputation parameters as needed for your specific AP-MS data. An example script for generating limma-based differential expression results, adapted to our dataset, can be found in the repository (https://github.com/Muedi/IRF4-in-Treg-and-Th17/tree/main/STAR/limma_analysis.R). This script includes essential steps and input data to reproduce the results presented in the STAR Protocol and associated figures.

### Combinatorial transcription factor motif analysis

**Timing: 1–2 days**

The present section describes how to annotate candidate regulatory regions with immune-specific TF binding sites using the Match algorithm from the TRANSFAC database^20^ following Bio-ChIP-seq peak calling: First, genomic DNA sequences corresponding to Bio-ChIP-seq peaks are extracted using the reference genome (e.g., mm10), and TRANSFAC configuration files referencing immune-specific TF profiles are prepared. These profiles can then be used to annotate motifs corresponding to immune regulators across all peak regions.

Subsequently, the output from the Match algorithm is parsed and allows to identify all instances where annotated motifs co-occur with another motif within <5 base pairs (bp) on the same strand, indicating potential synergistic regulatory interactions. The frequency of double-motif occurrences per peak region is then summarized to generate a dataset-wide overview of co-occuring TF motifs.

48. Download and index the reference genome.

wget http://hgdownload.soe.ucsc.edu/goldenPath/mm10/bigZips/mm10.fa.gz

gunzip mm10.fa.gz

samtools faidx mm10.fa

**CRITICAL:** Ensure that the genome build (e.g., mm10) matches the one used in peak calling.

49. Prepare peak sequences from Bio-ChIP-seq data (exported from EaSeq) in R.

library(readxl)

df <- read_excel("chip_th17.xlsx", .name_repair = "minimal")

df$Start <- df$Start - 1 # Convert to 0-based coordinates

df$Score <- 0 # Add placeholder score column

bed <- df[, c("Chromosome", "Start", "End", "Symbol", "Score", "Strand")]

write.table(bed, file = "chip_th17.bed", sep = "∖t", quote = FALSE, row.names = FALSE, col.names = FALSE)

***Note:*** Use the output from peak calling (exported from EaSeq) to generate a .bed file containing the genomic coordinates of Bio-ChIP-seq peaks. In this protocol, we use the file *chip_th17.xlsx* as an example input.

50. Extract peak sequences.

bedtools getfasta -fi mm10.fa -bed chip_th17.bed -fo chip_th17_peaks.fa -s

51. Prepare TRANSFAC matrix profile for immune specific TFs.

***Note:*** The modified profile applies a default cutoff of 0.8 for both matrix and core similarity scores, as this threshold provides a balance between sensitivity in detecting genuine binding sites and specificity in reducing false positives. These thresholds can be adjusted to modify the stringency as needed. You may also enhance this profile manually by adding any TFs of interest to ensure relevant motifs are included in the analysis.

awk 'NF==5 { $2="0.800"; $3="0.800"; print $0; next } { print }' ∖ TRANSFAC/match/data/prfs/immune_cell_specific.prf >∖

 TRANSFAC/match/data/prfs/immune_cell_specific_modified.prf

52. Create Match configuration .xml file named, e.g., *th17_config.xml* and insert the following.

<matchconfig maxHistogramRecordsNumber="100000" calcHistograms="false">

 <matrixLibs useCoreThreshold="true">

 <matrixLib URI="TRANSFAC/match/data/matrix.dat" ProfileURI="match/data/prfs/immune_cell_specific_modified.prf" scaled="true" />

 </matrixLibs>

 <output type="match" fileName="th17_immune_pulldown_match.out" useSegments="true" calcHistograms="false">

 <inputSequence URI="chip_th17_peaks.fa" name="" chr="" map="0" systemID="1" />

 </output>

</matchconfig>

***Note:*** Paths must be fully defined or relative to the execution location.

53. Run the TRANSFAC-Match algorithm.

cd TRANSFAC/match/bin/

./match.sh path/to/th17_config.xml

***Note:*** Set the correct path for the created *th17_config.xml.*

54. Convert Match output to structured .csv format.

input_file <- "th17_immune_pulldown_match.out"

output_file <- "th17_immune_pulldown_match.csv"

lines <- readLines(input_file)

header <- c("ID", "TF", "pos", "strand", "core_score", "matrix_score", "motif")

results <- list()

sequence_id <- NULL

for (line in lines) {

 if (grepl("Inspecting sequence ID", line)) {

 sequence_id <- trimws(tail(strsplit(line, " ")[[1]], 1))

 } else if (!is.null(sequence_id) && grepl("∖∖$", line)) {

 line_parts <- unlist(strsplit(line, " "))

 line_parts <- line_parts[line_parts != ""]

 tf <- line_parts[1]

 pos <- line_parts[3]

 strand <- line_parts[4]

 matrix_score <- line_parts[6]

 core_score <- line_parts[8]

 motif <- tail(line_parts, 1)

 row <- c(sequence_id, tf, pos, strand, core_score, matrix_score, motif)

 results <- append(results, list(row))

 }}

df <- as.data.frame(do.call(rbind, results), stringsAsFactors = FALSE)

colnames(df) <- header

write.csv(df, output_file,row.names = F)

55. Identify and annotate double-motif occurrences.

library(dplyr)

library(doParallel)

#Set number of cores

registerDoParallel(cores = 40)

df <- read.csv("th17_immune_pulldown_match.csv")

output <- foreach(id = unique(df$ID), .combine = rbind) %dopar% {

 id_df <- df %>% filter(ID == id) %>% arrange(pos)

 matches <- list()

 for (i in 1:(nrow(id_df) - 1)) {

 for (j in (i+1):nrow(id_df)) {

 if (id_df[i, "strand"] == id_df[j, "strand"]) {

 pos_diff <- abs(as.numeric(id_df[i, "pos"]) - as.numeric(id_df[j, "pos"]))

 if (pos_diff > 5) break

 matches[[length(matches) + 1]] <- data.frame(

  ID = id,

  strand = id_df[i, "strand"],

  pos_1 = id_df[i, "pos"],

  pos_2 = id_df[j, "pos"],

  motif_1 = id_df[i, "motif"],

  motif_2 = id_df[j, "motif"],

  TF1 = id_df[i, "TF"],

  TF2 = id_df[j, "TF"],

  stringsAsFactors = FALSE) }}}

 if (length(matches) > 0) do.call(rbind, matches) else NULL

}

stopImplicitCluster()

write.csv(output, "th17_combs.csv", row.names = FALSE)

***Note:*** This step is parallelized using 40 cores (adjust as available) to handle the large number of motif pairs generated when using comprehensive TF matrices. Parallelization significantly reduces runtime for dense motif datasets.

56. Count motif pair co-occurrences per region and save summary.

pairs_df <- read.csv("th17_combs.csv")

tf_pair_counts <- pairs_df %>%

 group_by(TF1, TF2) %>%

 summarise(ID_count = n_distinct(ID), .groups = "drop") %>%

 arrange(desc(ID_count))

write.csv(tf_pair_counts, "th17_combs_pair_counts.csv", row.names = FALSE)

## Expected outcomes

After raw MS data analysis, AP-MS experiments result in a list of proteins, including measured intensities, detected in your biotinylated and control samples. Further statistical analysis is required to determine the significance of interactors between two conditions as described in the step-by-step protocol using the limma package.^28^ The limma output allows to filter for significantly enriched proteins. We recommend to use an adjusted *p-value* for multiple hypothesis testing below 0.01 as well as an absolute fold change (FC) of >2 (i.e., log_2_(FC Bio/Ctrl) > 1 or higher) as filter criteria.

**Figure 2:**
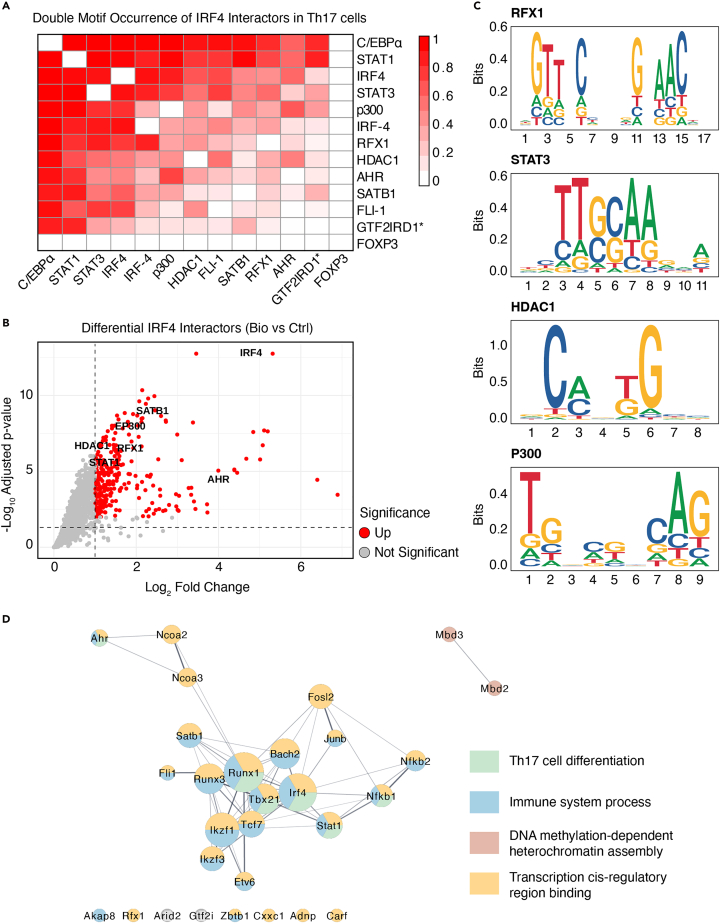
Integrated analysis of IRF4 interactors and double-motif occurrence (A) Double-motif occurrence of IRF4 interactors in Th17 ChIP-seq peaks. Heatmap shows normalized co-occurrence frequencies (0–1) for IRF4 together with transcription factors detected by AP-MS, considering only motifs occurring within <5 bp spacing in the same peak. Asterisk (∗): GTF2IRD1-isoform2. (B) Volcano plot of IRF4 interactors identified by AP-MS comparing biotinylated IRF4 (“Bio”) and control (“Ctrl”) conditions in Th17 cells. Labeled interactors indicate TFs with double-motif occurrences in Th17. (C) Sequence logos of the individual TF motifs used for double-motif annotation. (D) STRING-based IRF4 interaction network displaying interactors classified as TFs according to the criteria by Lambert *et al.*^38^ (STRING DB default settings). See also Table S2.

The motif annotation yields three output tables: a motif annotation table listing individual TF binding sites per Bio-ChIP-seq peak, a motif co-occurrence table identifying adjacent TF motifs on the same strand, and a summary table quantifying the number of motif pairs per region. Together, these results enable the identification of potential combinatorial transcriptional regulation. The output files generated can be used to investigate how many ChIP-seq peaks contain closely spaced (<5 bp) double-motifs for IRF4 and its identified interactors (Figure 2A; Table S1). These motif co-occurrence data can be cross-referenced with the significance of interactors identified by AP-MS (Figure 2B), highlighting candidates for potential cooperative or combinatorial binding in Th17 cells.

Furthermore, the individual motif logos (Figure 2C) provide an overview of which position-specific scoring matrices were used for motif annotation and help interpret which binding profiles contributed to the detection of double-motifs, especially when multiple motifs exist for a single TF.

In the present dataset, for example, a total of 29 IRF4 interactors are classified as TFs according to the criteria described by Lambert *et al.*^38^ (see Figure 2D; Table S2). Integrating data from combined motif and interactome analyses with transcriptome and proteome analyses of knock-out models—either of the TF of interest or its interactors—can help uncover underlying regulatory mechanisms and specific transcriptional programs driven by the TF complex of interest.

## Limitations

The present approach enables to identify PPIs at endogenous levels. However, it may require a large amount of input material depending on the expression levels of the protein of interest or when working with primary immune cells, such as T cells. Moreover, depending on the chosen *in vivo* biotinylation approach, preparatory work can be time-consuming, e.g., when establishing and breeding a suitable mouse model. Here, purchasing transgenic mice from companies offering respective services can help reduce the time requirements. Furthermore, precise binding mechanisms, e.g., site and mode of binding as well as structural details, cannot be discriminated by the presented approach and require additional follow-up experiments.

Efficient extraction and solubilization of the target protein of interest is another critical factor that has to be taken into consideration. As indicated in the step-by-step protocol, this can be addressed by testing and using different buffer formulations for nuclear lysis to maximize recovery of the target protein prior to the AP.

For the motif analysis, it has to be mentioned that motif co-occurrence should always be interpreted within the regulatory context of the TFs being studied, such as known binding partners and interaction mechanisms. While a close proximity of motifs may suggest competitive or cooperative binding, it does not confirm functional interaction. This limitation applies not only to IRF4 but to any TF analyzed using this approach, especially when multiple position-specific scoring matrices exist for TFs, capturing distinct binding modes.

## Troubleshooting

### Problem 1

Affinity-purified samples contain a high number of unspecific (background) proteins (related to Steps 2–9).

### Potential solutions

• Titrate different concentrations of the crosslinker to prevent over-crosslinking of the sample.
• Apply more stringent washing conditions during AP. This can be achieved by increasing salt and/or detergent concentration in the wash buffer or by adding additional washing steps with extended wash times.
• Perform cellular fractionation of the sample, e.g., isolate cell nuclei from cytoplasmic or membrane proteins, to reduce background signal and/or potential contaminating proteins, such as endogenous biotinylated proteins. Cellular fractionation can be visualized and analyzed by western blot. For this, it is recommended to use characteristic sub-cellular markers, such as lamin a/c for the nuclear fraction and actin for the cytoplasmic compartment, to identify optimal conditions.
• If cellular fractionation is already being applied, adjust the incubation time when adding the hypotonic buffer (i.e., a high number of cytosolic proteins is still present) or incubation conditions for this process.

### Problem 2

High signals of streptavidin are detectable in the MS data suppressing signals of co-eluting peptides (related to Steps 6–9).

### Potential solutions

• Optimize the conditions for protein elution from the streptavidin-coated beads. If working with a high number of samples, do the elution step in small batches to avoid prolonged incubation with the elution buffer.
• Use trypsin-resistant streptavidin beads (as for example, MagReSyn Streptavidin MS magnetic beads, https://resynbio.com/streptavidin/)

### Problem 3

Peptide recovery is low after proteolytic digestion (related to Steps 25–35).

### Potential solutions

• The protein amount isolated during AP may vary depending on expression levels of the bait protein of interest or input material used. Optimize and adjust trypsin amounts accordingly. Make sure that all beads are covered with the digestion solution. Sonicate samples to further promote disaggregation of beads prior to digestion. Digestion efficiency can be further improved by regularly shaking the samples during overnight digestion (e.g, with a ThermoMixer at 1,000 r.p.m.).^32^
• Ensure that the pH of your digestion and elution solutions is within the suitable range (pH 7–9).
• If no additional additives, such as detergents, are added to the trypsin solution, omit the peptide clean-up step to avoid sample losses during peptide purification. After tryptic digest, centrifuge samples at 20,000 × *g* for 1 min at 21°C–24°C. Place tubes be on a magnetic rack until the beads have settled, collect and transfer peptide containing supernatants into a fresh tube.

### Problem 4

Access to TRANSFAC profiles is license-restricted (related to Step 51).

### Potential solution

The motif annotation scripts provided in this STAR Protocol are adapted for use with TRANSFAC matrices. Users without a TRANSFAC license may instead employ the Match-Suite^39^ with open-access motif collections such as JASPAR^40^ (https://jaspar.elixir.no/).

### Problem 5

The immune-specific TRANSFAC profile file may not cover all TFs relevant to the biological system of interest (related to Step 51).

### Potential solution

Within the TRANSFAC installation directory *match/data/prfs*, multiple profile files are provided, including *vertebrate.prf*, which contains a broad set of TFs. These .prf files list abbreviations (e.g., *V$IRF4_01*) along with their associated matrix and core similarity default scores. Browse these files to identify TFs of interest and include them in the custom profile.

### Problem 6

Too many motif matches are detected, including likely false positives, which obscures meaningful results (related to Step 51).

### Potential solution

Increase the core similarity threshold to focus on the most conserved positions within each motif. You may also moderately raise the matrix similarity threshold to further restrict motif matches to higher-confidence binding sites.

### Problem 7

Many detected motif co-occurrences are the result of substantial overlap between the two motifs, often reflecting near-identical binding sites (related to Step 55).

### Potential solution

Since multiple matrices often exist for the same TF (e.g., *V$IRF4_01, V$IRF4_02*), each representing different experimental sources or binding contexts, it is important not to rely on a single position-specific scoring matrix when inferring TF co-binding. Investigate whether the observed co-occurrence holds true across other matrix combinations representing the same TFs. Consistent co-binding across multiple matrix pairs increases confidence in the biological relevance of the interaction and reduces the chance that the result is an artifact of one specific matrix.

## Resource availability

### Lead contact

Requests for further information and resources and reagents should be directed to and will be fulfilled by the lead contact, Ute Distler (ute.distler@uni-mainz.de).

### Technical contact

Technical questions on executing this protocol should be directed to and will be answered by the technical contacts, Anna Gabele (angabele@uni-mainz.de), Mert Cihan (mercihan@uni-mainz.de), Ute Distler (ute.distler@uni-mainz.de) or Tobias Bopp (boppt@uni-mainz.de).

### Materials availability

This study did not generate any new unique reagents.

### Data and code availability

• The R-scripts used for statistical and Bio-ChIP-seq analysis are made available in this protocol. Extended scripts for limma analysis as used in Gabele *et al.*^1^ can be accessed on https://github.com/Muedi/IRF4-in-Treg-and-Th17 and https://doi.org/10.5281/zenodo.14643315. The script used for the present manuscript can be accessed under https://github.com/Muedi/IRF4-in-Treg-and-Th17/tree/main/STAR
• The published article, Gabele *et al.*,^1^ includes all datasets analyzed during this study. They can be accessed on the following repositories: The mass spectrometry proteomics data is deposited on ProteomeXchange as well as on jPOST with the dataset identifiers PXD044298/JPST002260. The Bio-ChIP-seq data can be accessed on gene expression omnibus (GEO) via the accession number GSE240979.

## Acknowledgments

This work was supported by the German Research Foundation (DFG; project no. 318346496, SFB1292/2 TP01 to T.B., TP11 to U.D. and N.L., and TP-Q1 to S.T. as well as DI 2471/1-1 to U.D. and BO 3306/2-1 to T.B.); the DFG priority program SPP 2225 (project no. 446605368 to U.D.); SFB TRR355 (TPA9 and TPA10 to T.B.); the German Federal Ministry of Research, Technology and Space (BMFTR, DIASyM,FKZ 03LW0241K to S.T.); and the Research Center for Immunotherapy (FZI) of the Johannes Gutenberg-University Mainz.

## Author contributions

A.G. optimized the AP-MS protocol, performed Bio-ChIP-seq analysis as well as preparatory steps (peak calling) for motif analysis (ChIP-seq), and prepared the initial draft of the manuscript. M.C. wrote the code for motif analysis and prepared the initial draft of the manuscript. M.S. contributed to the conceptualization of bioinformatic analyses and wrote the code for limma analysis. M.K. assisted with the ChIP-seq analyses and analyzed ChIP-seq datasets. A.N. and K.R. assisted with the wet lab experiments. M.A.A.-N. contributed to the conceptualization of bioinformatic analyses. N.L. and S.T. acquired funding and contributed to the conceptualization of AP-MS experiments. T.B. acquired funding and conceptualized and designed the project. U.D. acquired funding, conceptualized and designed the project, optimized the AP-MS protocol, conducted MS analyses, and prepared the initial draft of the manuscript. All authors discussed and reviewed the final manuscript version.

## Declaration of interests

The authors declare no competing interests.

## Supplemental information

Table S1. List of significant IRF4 interactors in murine CD4+ T cells differentiated under Th17-skewing conditions as reported after limma analysis, related to the section “expected outcomes” The table provides the limma output. A detailed description can be also found in the respective documentation (https://bioconductor.org/packages/release/bioc/html/limma.html).

Table S2. List of significant IRF4 interactors in murine CD4+ T cells differentiated under Th17-skewing conditions as reported after limma analysis filtered for proteins that are classified as transcription factors according to the criteria by Lambert et al. (https://doi.org/10.1016/J.CELL.2018.01.029), related to the section “expected outcomes” and source data for network displayed in Figure 2D
